# Modeling Bainbridge-Ropers Syndrome in *Xenopus laevis* Embryos

**DOI:** 10.3389/fphys.2020.00075

**Published:** 2020-02-18

**Authors:** Hava Lichtig, Artyom Artamonov, Hanna Polevoy, Christine D. Reid, Stephanie L. Bielas, Dale Frank

**Affiliations:** ^1^Department of Biochemistry, Faculty of Medicine, The Rappaport Family Institute for Research in the Medical Sciences, Technion – Israel Institute of Technology, Haifa, Israel; ^2^Department of Genetics, Stanford University, Stanford, CA, United States; ^3^Department of Human Genetics, University of Michigan Medical School, Ann Arbor, MI, United States

**Keywords:** Bainbridge-Ropers syndrome, ASXL3 protein, *Xenopus laevis* embryos, neurodevelopmental disease model, neural crest

## Abstract

The *Additional sex combs-like (ASXL1-3)* genes are linked to human neurodevelopmental disorders. The *de novo* truncating variants in ASXL1-3 proteins serve as the genetic basis for severe neurodevelopmental diseases such as Bohring-Opitz, Shashi-Pena, and Bainbridge-Ropers syndromes, respectively. The phenotypes of these syndromes are similar but not identical, and include dramatic craniofacial defects, microcephaly, developmental delay, and severe intellectual disability, with a loss of speech and language. Bainbridge-Ropers syndrome resulting from *ASXL3* gene mutations also includes features of autism spectrum disorder. Human genomic studies also identified missense *ASXL3* variants associated with autism spectrum disorder, but lacking more severe Bainbridge-Ropers syndromic features. While these findings strongly implicate *ASXL3* in mammalian brain development, its functions are not clearly understood. ASXL3 protein is a component of the polycomb deubiquitinase complex that removes mono-ubiquitin from Histone H2A. Dynamic chromatin modifications play important roles in the specification of cell fates during early neural patterning and development. In this study, we utilize the frog, *Xenopus laevis* as a simpler and more accessible vertebrate neurodevelopmental model system to understand the embryological cause of Bainbridge-Ropers syndrome. We have found that ASXL3 protein knockdown during early embryo development highly perturbs neural cell fate specification, potentially resembling the Bainbridge-Ropers syndrome phenotype in humans. Thus, the frog embryo is a powerful tool for understanding the etiology of Bainbridge-Ropers syndrome in humans.

## Introduction

Bainbridge-Ropers syndrome (BRS; OMIM 615485) is characterized by failure to thrive, craniofacial defects, feeding problems, global developmental delay, hypotonia, intellectual disability and delays in language acquisition (Bainbridge et al., 2013; Russell and Graham, 2013). *De novo* truncating mutations in *Additional sex combs-like 3* (*ASXL3*) have emerged as the cause of BRS, while missense mutations in ASXL3 have been identified in individuals with autism spectrum disorder (ASD) (Dinwiddie et al., 2013; Russell and Graham, 2013; De Rubeis et al., 2014). Many *de novo* mutations disrupt chromatin remodeling genes, suggesting that chromatin perturbation and the subsequent misregulation of transcription is an important molecular mechanism in human genetic disease (Veltman and Brunner, 2012; Allen et al., 2013; Dinwiddie et al., 2013; Ku et al., 2013). *ASXL* genes are vertebrate homologs of the *Drosophila Additional sex combs* gene. Members of this gene family enhance transcription regulation by Polycomb-group and Trithorax-group complexes (Gaytán de Ayala Alonso et al., 2007; Baskind et al., 2009; Fisher et al., 2010). In vertebrates, the ASXL family consists of three members, the ASXL1, ASXL2, and ASXL3 proteins (Fisher et al., 2006; Aravind and Iyer, 2012; Gelsi-Boyer et al., 2012; Katoh, 2013, 2015). Germline mutations of ASXL proteins occur in patients with congenital disorders, whereas somatic mutations are seen in cancer (Gelsi-Boyer et al., 2012; Katoh, 2013; Srivastava et al., 2017).

Molecular functions have been predicted for ASXL3 based on its similarity to other ASXL family proteins, but its true function remains unknown. ASXL1 protein acts as a scaffold for ubiquitin C-terminal hydrolase (BAP1), histone lysine methyltransferase (EZH2) and nuclear receptors (Katoh, 2015). ASXL1 interacts with BAP1 protein to form the human Polycomb repressive deubiquitination (PR-DUB) complex, which removes mono-ubiquitin from lysine 119 of histone H2A (Di Croce and Helin, 2013). BAP1 alone cannot deubiquitinate H2A, so PR-DUB formation is critical for normal function. While BAP1 is a required component of a PR-DUB, other ASXL family members in addition to ASXL1 are likely interchangeable to form the active deubiquitinase complex (Scheuermann et al., 2010; Lai and Wang, 2013; Srivastava et al., 2016). In human cells, ASXL3 protein was shown to interact with BAP1 to form the PR-DUB complex (Srivastava et al., 2016).

In primary fibroblasts established from a BRS patient with a truncation mutation, *asxl3* mRNA underwent nonsense mediated decay, suggesting that BRS results from a reduction in ASXL3 activity (Srivastava et al., 2016). In these fibroblasts, H2A ubiquitination was increased, suggesting that either the reduced levels of endogenous ASXL3 protein and/or the ASXL3 truncated protein disrupt the normal PR-DUB complex activity. In primary human cells, the effect of elevated histone H2 ubiquitination on global transcription was examined by comparing gene expression levels in ASXL3 truncation versus normal individuals. This study found over five hundred genes had significant changes in expression in BRS derived cells versus controls, half increased while half decreased (Srivastava et al., 2016). Interestingly, the truncated ASXL3 protein interacts with BAP1 protein (Srivastava et al., 2016). Germline mutations in PRC1 complex components were shown to disrupt H2A mono-ubiquitination in ASD and primary microcephaly (Awad et al., 2013; Gao et al., 2014). BRS is the first single gene disorder shown to exhibit defects in deubiquitination of H2A (Srivastava et al., 2016). These findings highlight a role for dynamic regulation of H2A ubiquitination in neurodevelopmental disease.

Often, in human genetic disease, the path from mutation to function is not an easy one. Because of the complexities of human neurodevelopment, it remains unclear how the ASXL3 truncation mutation leads to BRS. Truncated ASXL3 protein could be dominant-negative, hypomorphic or dead. Not mutually exclusive, the protein could be functional (varying degrees of hypomorphy) and its activity could be rate-limited due to RNA mediated decay and overall reduced ASXL3 protein levels causing a haploinsufficiency phenotype. Understanding the correct temporal role of ASXL3 protein in early neural development will elucidate mechanisms underlying both BRS and autism syndromes caused by ASXL3 activity perturbation in early human embryonic development. Early *Xenopus* development offers an easy manipulable system for unraveling the etiology of the ASXL3 induced disease state. *Xenopus* is unique in offering the resolution of a changing organism with a disease phenotype/state and not a cell line to examine these questions. We examine the effects of ASXL3 knockdown through all stages of early neurodevelopment in a whole organism. We have found that knockdown of ASXL3 protein disturbs the earliest stages of neural cell fate specification, including early nervous system induction and anteroposterior (AP) patterning. ASXL3 morphant embryos express lower levels of genes required for correct formation of forebrain, hindbrain, primary neurons and neural crest. Analysis of patients and fetuses of BRS patients show similar defects in craniofacial, motor neuron and hindbrain development (Bainbridge et al., 2013; Balasubramanian et al., 2017; Bacrot et al., 2018). These results show that perturbation of ASXL3 function during early vertebrate development has devastating effects on formation of the early nervous system.

## Results

### ASXL3 Knockdown Modulates Neural Cell Fate Formation

**FIGURE 1:**
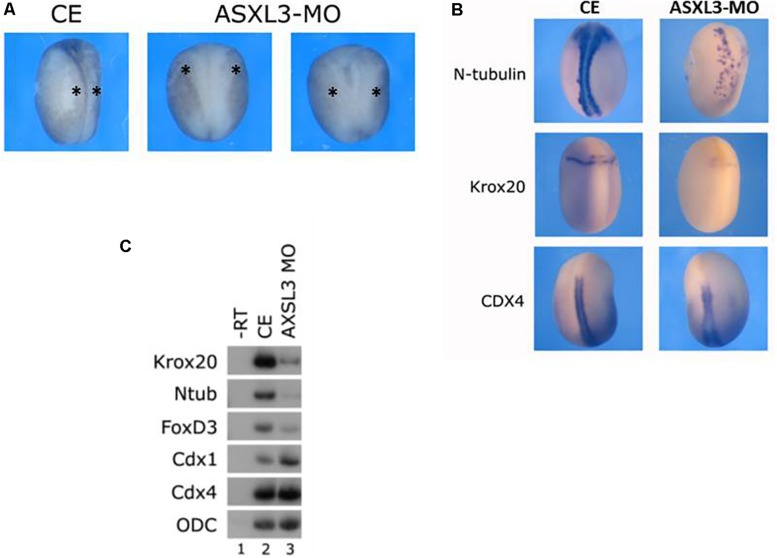
ASXL3 knockdown causes neural tube defects and inhibits expression of posterior neural markers. **(A)** Neurula stage embryos were examined for neural tube defect phenotypes. Control embryos (CE – left panel) were injected with 15ng *ASXL3-MO* at the one cell stage (two right panels) The *ASXL3* morphant embryos are squat, less elongated, with poor neural folding and open neural plates. In the CE group 87% of the embryos had normal neural folds (*n* = 158) versus the *ASXL3-MO* groups in which 86% had an open neural plate phenotype (*n* = 603). This was repeated in six independent experiments. The star marks the neural folds regions in the embryos. **(B)** Control embryos (CE, left panel) were injected at the one-cell stage with *ASXL3-MO* (15 ng, right panel). *In situ* hybridization was carried out on late neurula (st.18) embryos to the markers: *n-tub* (primary neuron), *krox20* (hindbrain and lateral neural crest stripes) and *cdx4* (spinal cord). *Ntub* was weakly expressed in 68% (*n* = 19) of the embryos; *krox20* was weakly expressed in 85% (*n* = 20) of the embryos and *cdx4* expression was normal in 81% (*n* = 21) of the *ASXL3-MO* injected embryos. **(C)** Control embryos (lane 2) were injected at the one-cell stage with *ASXL3-MO* (15 ng, lane 3). sqRT-PCR was carried out on total RNA isolated from pools of eight neurula (st.16) embryos from each group to the genes: *krox20* (hindbrain) *ntub* (primary neuron), *foxd3* (neural crest) and *cdx1/4* (spinal cord). -RT was performed (lane 1) to total RNA isolated from the control uninjected embryos. The housekeeping gene *ODC* serves as positive control for RNA levels.

To determine a role for ASXL3 protein during early *Xenopus* development, we depleted endogenous ASXL3 protein by using a translation blocking morpholino oligonucleotide (MO). ASXL3 knockdown embryos show a modulated posterior neural structure, impeded neural plate elongation, with slowed and reduced neural folding (Figure 1A, see star). To characterize this phenotype in neurula stage embryos, neural markers were examined by both *in situ* hybridization (Figure 1B, compare left to right panels) and sqRT-PCR (Figure 1C, compare lanes 2–3). In ASXL3 knockdown embryos, expression of hindbrain (*krox20*), neural crest (*foxd3*), and primary neuron (*n-tub*) markers was severely reduced. The number of primary neurons was lower and their normal arrangement was also perturbed, as observed by *n-tub* expression (Figure 1B). Interestingly spinal cord marker (*cdx*) expression was not reduced (Figures 1B,C); *cdx1* expression was consistently increased slightly in ASXL3 morphant embryos (Figure 1C, compare lanes 2–3).

These results suggest that ASXL3 protein is a crucial regulator that balances the correct levels of early posterior neural cell fates. These observations also suggest that ASXL3 may function in the earliest network of genes regulating the specification of posterior-trunk neural tissue such as hindbrain, primary neurons and neural crest. This phenotype is quite similar to the knockdown phenotype for the Meis3 homeodomain protein (Dibner et al., 2001; Gutkovich et al., 2010) or embryos disrupted for early Wnt-signaling, where hindbrain, neural crest, and primary neuron cell fates are lost, but the spinal cord forms quite normally (Elkouby et al., 2010; Polevoy et al., 2019).

### Ectopic Expression of Full-Length or Truncated Human ASXL3 Protein Rescues the Knockdown Phenotype

**FIGURE 2:**
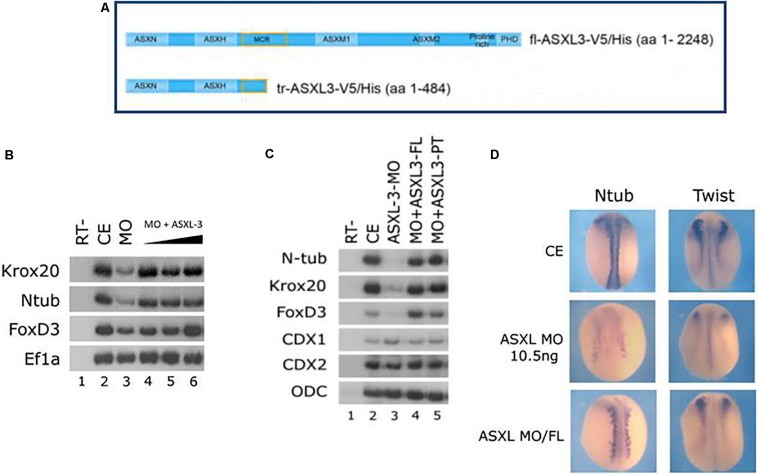
Full-length and truncated/mutant human ASXL3 proteins rescue the *ASXL3-MO* Knockdown phenotype. **(A)** A schematic illustration of the main functional units of the full-length human and truncated/mutant ASXL3 proteins that were used in this study (Srivastava et al., 2016). **(B)** Control embryos (lane 2) were injected at the one-cell stage with *ASXL3-MO* (12.5 ng, lane 3) and increasing concentrations of the full-length mRNA (250–750 pg) encoding the human ASXL3 protein (lanes 4–6). sqRT-PCR was carried out on total RNA isolated from pools of seven neurula (st.16) embryos to the genes: *krox20*, *ntub*, and *foxd3*. -RT was performed (lane 1) to RNA isolated from the uninjected control embryos. The housekeeping gene *Ef1*α serves as positive control for RNA levels. **(C)** Control embryos (lane 2) were injected at the one-cell stage with *ASXL3-MO* (12.5 ng, lane 3) and the mRNAs (500 pg) encoding the full-length (FL – 2248 amino acids) or truncated/mutant (PT – 484 amino acids) human ASXL3 proteins (lanes 4–5). sqRT-PCR was carried out on total RNA isolated from pools of seven neurula (st.17–18) embryos to the genes: *ntub*, *krox20*, *foxd3*, *cdx1*, and *cdx2*. -RT was performed (lane 1) to RNA isolated from the uninjected control embryos. The housekeeping gene *ODC* serves as positive control for RNA levels. **(D)** Control embryos (CE, top panels) were injected at the one-cell stage with *ASXL3-MO* (middle panels) and mRNA (300 pg) encoding the full-length (FL) human ASXL3 protein (bottom panels). *In situ* hybridization was carried out in late neurula stage embryos to the markers: *ntub* (primary neuron) and twist (neural crest). *Ntub* was weakly expressed in 81% (*n* = 21) of the *ASXL3-MO* embryos versus controls (compare top/middle panels, left side); rescued expression was observed in 68% (*n* = 19) of the *ASXL3-MO* embryos (compare bottom/middle panels, left side). *Twist* was weakly expressed in 90% (*n* = 21) of the *ASXL3-MO* embryos versus controls (compare top/middle panels, right side); rescued expression was observed in 46% (*n* = 24) of the *ASXL3-MO* embryos (compare bottom/middle panels, right side).

The wild-type full-length (FL) human ASXL3 protein (Figure 2A) functions during early *Xenopus* development. Ectopic expression of human ASXL3 protein rescued neural marker expression in ASXL3 morphant embryos (Figure 2B, compare lanes 3–6). As shown by *in situ* hybridization (Figure 2D), primary neuron (*ntub*, left panel) and neural crest (*twist*, right panel) marker expression is significantly rescued, more so resembling the normal uninjected embryos for both levels and pattern of gene expression. Ectopic expression of ASXL3 in normal embryos at the concentration described (Figures 2B,C) does not alter gene expression of neural markers or cause a significant phenotype (not shown). We also ectopically expressed a mutant/truncated form of the ASXL3 protein (Figure 2A) associated with BRS into ASXL3 morphant embryos (Figure 2C). This mutant ASXL3 protein has a severe truncation, having an ORF of 484 amino acids in comparison to the wildtype ASXL protein of 2248 amino acids (Figure 2A). This mutant protein has a functional N-terminal domain that interacts with the BAP1 hydrolase protein that removes mono-ubiquitin from histone H2A lysine 119 as a component of the PR-DUB complex, like wild type ASXL3 protein (Srivastava et al., 2016). Somewhat surprisingly, when compared to the wildtype protein, the truncated ASXL3 protein also robustly rescued neural marker expression in the morphant embryos (Figure 2C, compare lanes 3–5). This result suggests that the truncated protein has significant biological activity via its forced ectopic expression in *Xenopus* embryos. In BRS, it was suggested that nonsense mediated decay may affect *asxl3* mRNA and subsequent protein levels (Srivastava et al., 2016). This is a dilemma for human geneticists in trying to understand the etiology and mechanism of disease. However, in our assay of ectopic forced over-expression, the protein seems not to be dead or dominant-negative, but fairly functional.

### ASXL3 Is Necessary to Induce Posterior Neural Cell Fates in Embryonic Explants

**FIGURE 3:**
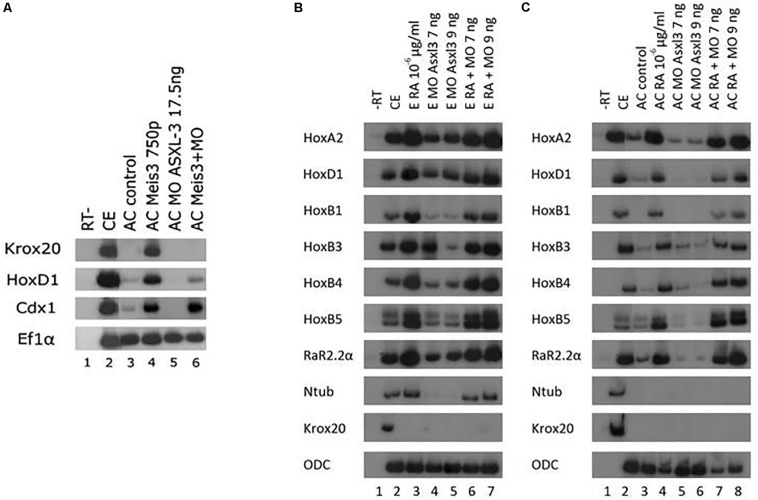
ASXL3 is necessary to induce posterior neural cell fates via Meis3 but not via retinoic acid. **(A)** One-cell stage embryos (lane 2) were injected at the one-cell stage with *meis3* encoding RNA (750 pg, lane 4), *ASXL3-MO* (17.5 ng, lane 5) or both (lane 6). AC explants were removed from control (lane 3) and injected embryos (lanes 4–6) at blastula stage 9, and cultured to neurula stage 16. sqRT-PCR was carried out on total RNA isolated from five control embryos (lane 2), and from eighteen ACs from each group (lanes 3–6) to the genes: *krox20, hoxd1* (hindbrain), *cdx1* (spinal cord). -RT was performed for RNA levels. The housekeeping gene *Ef1*α serves as positive control for RNA levels. **(B)** Control embryos (lane 2) were injected at the one-cell stage with *ASXL3-MO* (7 and 9 ng, lanes 5 and 6, respectively). Upon reaching blastula stage 10 control embryos and the injected groups were placed in retinoic acid at 10^–6^ μg/ml (lanes 3, 6, 7). The Embryos were cultured to neurula stage 16. sqRT-PCR was carried out on total RNA isolated from five control embryos (lane 2), and from eighteen ACs from each group (lanes 3–8) to the genes: *hoxa2, hoxb1, hoxb3, hoxb4, hoxd1, rar2.2*α, *krox20* (hindbrain), *hoxb5* (spinal cord), and *ntub* (primary neuron). -RT was performed for RNA levels. The housekeeping gene *ODC* serves as positive control for RNA levels. **(C)** Control embryos (lane 2) were injected at the one-cell stage with *ASXL3-MO* 7 and 9 ng, (lanes 5 and 6, respectively). AC explants were removed from control (lane 3) and injected embryos (lanes 3–8) at blastula stage 9, and upon reaching blastula stage 10 AC controls and the injected groups were placed in retinoic acid at 10^–6^ μg/ml (lanes 4, 7, 8). The ACs were cultured to neurula stage 16. sqRT-PCR was carried out on total RNA isolated from five control embryos (lane 2), and from eighteen ACs from each group (lanes 3–8) to the genes: *hoxa2, hoxb1, hoxb3, hoxb4, hoxd1, rar2.2*α, *krox20* (hindbrain), *hoxb5* (spinal cord), and *ntub* (primary neuron). -RT was performed for RNA levels. The housekeeping gene *ODC* serves as positive control for RNA levels.

*Xenopus* offers an opportunity to use explant assays in real embryonic time to directly study induction of cell fate specification in pluripotential cells. We have utilized the animal cap (AC) assay to determine potential pathway interactions for ASXL3 in the embryo. We initially addressed ASXL3 interactions with the Meis3 homeodomain protein, since this protein is one of the most important early regulators of posterior neural cell fates (Dibner et al., 2001). In Meis3 morphant embryos, there is a loss of posterior neural cell fates (Dibner et al., 2001; Gutkovich et al., 2010), similar to the ASXL3 knockdown (Figures 1B,C, 2B,C). To address this question, we co-injected one-cell stage embryos with mRNA encoding Meis3 protein and the *ASXL3-MO*. AC explants were removed at blastula stages and cultured to neurula stages, and posterior neural marker expression was examined. In this AC assay (Salzberg et al., 1999), Meis3 robustly induces expression (Figure 3A, compare lanes 3–4) of hindbrain (*krox20*, *hoxd1*) and spinal cord markers (*cdx1*). Co-expression of Meis3/*ASXL3-MO* led to a sharp decrease in hindbrain marker (*krox20*, *hoxd1*) expression, while the *cdx1* spinal cord marker expression levels were virtually unchanged (Figure 3A, compare lanes 4 and 6) or slightly increased. Thus in the absence of ASXL3, Meis3 protein is differentially perturbed, it cannot induce hindbrain but still efficiently activates expression of spinal cord markers. These results mimic the observation seen in ASXL3 morphant embryos where hindbrain formation is perturbed, but the spinal cord marker expression is fairly normal (Figures 1B,C). This result suggests that ASXL3 acts in parallel with Meis3 protein in hindbrain specification.

Retinoic acid (RA) signaling is crucial for formation and patterning the hindbrain during early embryonic development (rev. in Frank and Sela-Donenfeld, 2019). We examined if ASXL3 knockdown modified RA inducing activity in embryos or AC explants. Embryos were treated with RA at gastrula stages and examined for neural markers at neurula stages. Expression levels of genes activated by RA in embryos or explants, such as the hindbrain markers *hoxa2, hoxd1, hoxb1, hoxb3, hoxb4, rar2.2*α *krox20*, spinal cord marker *hoxb5*, were examined (Figures 3B,C). In the ASXL3 morphant embryos, there was a strong reduction in the expression of the *hoxb1, hoxb3*, and *krox20* hindbrain markers. An intermediate inhibitory effect was observed for the hindbrain markers *hoxa2* and *rar2.2*α. No significant inhibition was seen in ASXL3 morphant embryos for the more posterior *hoxb4* (hindbrain/spinal cord border) and *hoxb5* (spinal cord) expression. All of these markers are activated by RA, except for *krox20* which is repressed by both the *ASXL3-MO* and RA treatment (Dibner et al., 2004). In the *ASXL3-MO*/RA group, the genes repressed by the *ASXL3-MO* are re-activated to high levels by RA, similar to control embryos. Clearly RA canceled the negative effect on gene expression mediated by ASXL3 protein knockdown. Similar results were seen in animal caps, in which the expression of RA target genes was not inhibited by the *ASXL3-MO* (Figure 3C, compare lanes 3–4 to 7–8). Thus RA signaling seems to act independently of ASXL3 protein activity during posterior neural cell fate specification.

### Inhibition of ASXL3 Differentially Modifies Noggin Neural Inducing Activity

**FIGURE 4:**
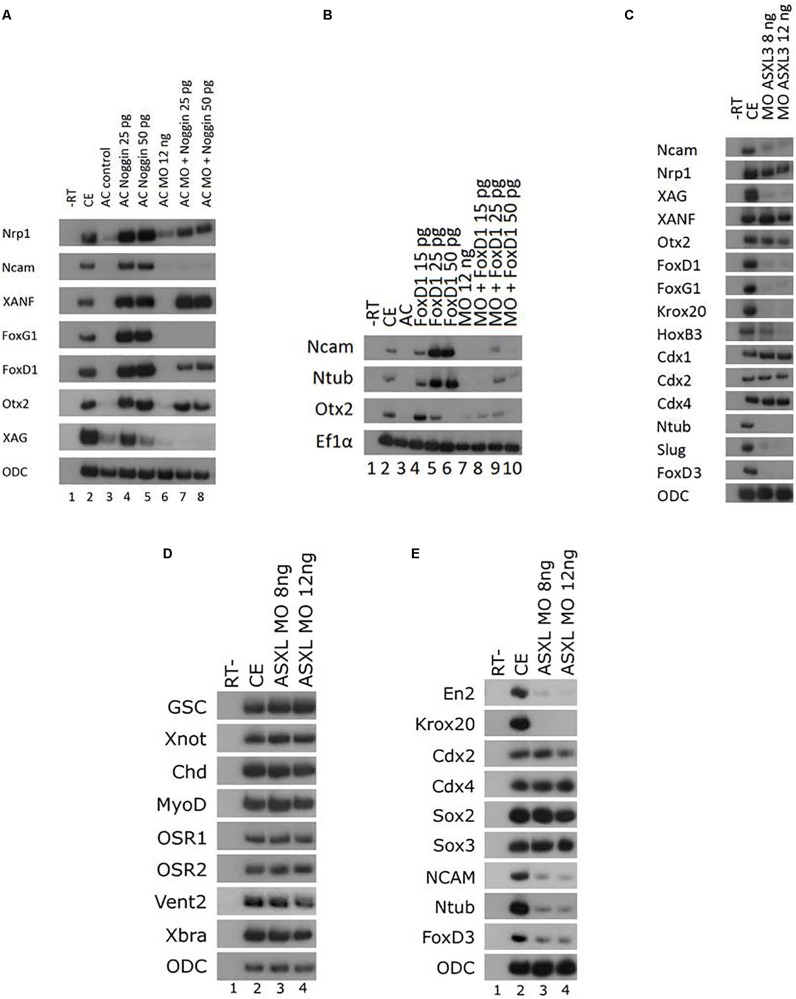
Inhibition of ASXL3 differentially modifies noggin neuralizing activity. **(A)** Control embryos (lane 2) were injected at the one-cell stage with noggin encoding RNA (25 and 50 pg, lanes 4 and 5), *ASXL3-MO* (12 ng, lane 6), or both (lanes 7 and 8). AC explants were removed from control (lane 3) and injected embryos (lanes 4–8) at blastula stage 9, and cultured to neurula stage 16. sqRT-PCR was carried out on total RNA isolated from five control embryos (lane 2), and from eighteen ACs from each group (lanes 3–8) to the genes: *nrp1, ncam* (panneural), *xanf1, foxg1, foxd1, otx2* (forebrain), and *xag* (cement gland). -RT was performed for RNA levels. The housekeeping gene *ODC* serves as positive control for RNA levels. **(B)** Control embryos (lane 2) were injected at the one-cell stage with *ASXL3-MO* (12 ng, lane 7) and mRNA (15, 25, 50 pg) encoding foxd1 (lanes 4–6), or both (lanes 8–10). AC explants were removed from control (lane 3) and injected embryos (lanes 4–10) at blastula stage, and cultured to neurula stage 16. sqRT-PCR was carried out on total RNA isolated from five control embryos (lane 2), and from eighteen ACs from each group (lanes 3–10). sqRT-PCR was carried out on total RNA isolated from pools of seven neurula (st.16) embryos to the genes: *ncam* (panneural), *ntub* (primary neurons), and *otx2* (forebrain). -RT was performed (lane 1) to RNA isolated from the uninjected control embryos. The housekeeping gene *Ef1*α serves as positive control for RNA levels. **(C)** Control embryos (lane 2) were injected at the one-cell stage with *ASXL3-MO* (8, 12 ng; lanes 3–4). sqRT-PCR was carried out on total RNA isolated from pools of five neurula (st.16) embryos from each group to the genes: *ncam*, *nrp1, xag*, *xanf*, *otx2*, *foxd1*, *foxg1*, *krox20*, *hoxb3*, *cdx1*, *cdx2*, *cdx4*, *ntub*, *snail2*, and *foxd3*. -RT was performed (lane 1) to total RNA isolated from the control uninjected embryos. The housekeeping gene *ODC* serves as positive control for RNA levels. **(D)** Control embryos (lane 2) were injected at the one-cell stage with *ASXL3-MO* (8, 12 ng; lanes 3–4). sqRT-PCR was carried out on total RNA isolated from pools of six gastrula (st.11.5) embryos from each group to the genes: *goosecoid* (*gsc*), *xnot*, *chordin* (*chd*), *myod*, *osr1*, *osr2*, *vent2*, and *brachyury* (*xbra*). -RT was performed (lane 1) to total RNA isolated from uninjected control embryos. The housekeeping gene *ODC* serves as positive control for RNA levels. **(E)** Sibling embryos from **(D)** were taken for sqRT-PCR. Total RNA was isolated from pools of six neurula (st.16) embryos to the genes: *engrailed2* (*en2*), *krox20*, *cdx2*/*4*, *sox2*/*3*, *ncam*, *ntub*, and *foxd3*. -RT (lane 1) to RNA isolated from the control embryos. The housekeeping gene *ODC* serves as positive control for RNA levels.

To further explore the role of ASXL3 protein in early neural development, we examined if initial anterior and general neural induction by a BMP antagonist (noggin) or one of its downstream mediators requires functional ASXL3 protein. We co-expressed the BMP-antagonist protein noggin and the *ASXL3-MO* in ACs (Figure 4A). Noggin alone robustly induces panneural (*nrp1*, *ncam*), cement gland (*xag*) and forebrain marker (*xanf*, *foxd1*, *foxg1*, *otx2*) expression in ACs (Figure 4A, compare lanes 3–5). In Noggin/*ASXL3-MO* co-expressing ACs, neural markers were not all inhibited to the same extent. The *ncam*, *foxd1*, *foxg1*, and *xag* markers were the most strongly inhibited (Figure 4A, compare lanes 3–5 to lanes 7–8). Moderate inhibition was observed for the *otx2* and *nrp1* markers (Figure 4A, compare lanes 3–5 to lanes 7–8), while expression of the most anterior *xanf1* marker was slightly stimulated (Figure 4A, compare lanes 3–5 to lanes 7–8). This observation suggests that ASXL3 is not acting as a simple permissive on/off switch for noggin activity. ASXL3 protein appears to be modifying noggin neural activity in a complex manner that may have implications for cell fate specification in the developing embryo. Interestingly, expression of the most anterior *xanf1* forebrain marker was slightly stimulated and not repressed by ASXL3 knock down (Figure 4A). Ectopic XANF1 protein levels were shown to repress expression of the adjacently expressed *otx2* and *foxg1* forebrain markers (Ermakova et al., 1999). Perhaps ASXL3 knockdown sets the stage for more efficient repression of forebrain genes by XANF1, by increasing its expression levels at the cost of more posterior forebrain regions.

ASXL3 also regulates gene expression downstream to noggin. To address this point, we examined the effect of ASXL3 knockdown on FoxD1 protein activity. FoxD1 is a transcriptional repressor protein, whose expression is induced by BMP-antagonism (noggin) in *Xenopus*, and it acts downstream to induce expression of panneural and forebrain markers (Mariani and Harland, 1998; Polevoy et al., 2017). FoxD1 is required as a mediator of early neural induction in the forebrain region in *Xenopus* (Mariani and Harland, 1998; Polevoy et al., 2017). In ACs, FoxD1 induces expression of a primary neuron marker (*ntub*) in a Wnt-dependent manner (Fonar et al., 2011; Polevoy et al., 2017). In ACs, FoxD1 protein induced panneural (*ncam*), forebrain (*otx2*) and *ntub* marker expression (Figure 4B, compare lanes 3–6). In FoxD1/*ASXL3-MO* co-expressing animal caps, similar to noggin, FoxD1 fails to efficiently induce these markers (Figure 4B, compare lanes 4–6 to lanes 8–10). Thus, activity of the BMP-antagonism downstream mediator, FoxD1 is inhibited by the absence ASXL3 protein. Interestingly, *ntub* expression is also inhibited, suggesting that Wnt-dependent processes are also affected. This result complements our observation in ASXL3 morphant embryos, where Wnt-dependent cell fates such as primary neurons, hindbrain and neural crest are all reduced (Figures 1B,C).

**FIGURE 5:**
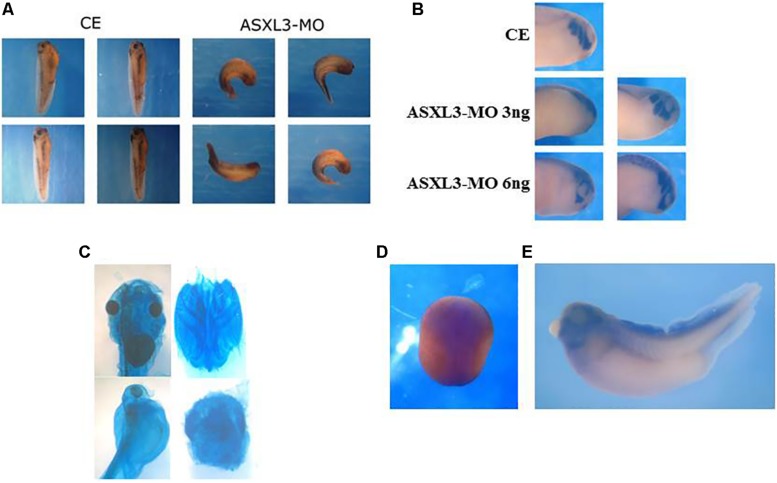
ASXL3 knockdown disrupts head formation. **(A)** Stage 37 tadpole stage embryos were examined for axial defect phenotypes. Control embryos (CE – four left panels) were injected with 12 ng *ASXL3-MO* at the one-cell stage (four right panels) The *ASXL3* morphant embryos are squat, less elongated, with poor trunk and head development. In the CE group 100% of the embryos developed normally development (*n* = 41) versus the *ASXL3-MO* group in which 100% of the embryos had a disrupted axial phenotype (*n* = 41). Four representative embryos are shown for each group. **(B)** Embryos were microinjected with *ASXL3-MO* (3 or 6 ng) into one-blastomere at the two-cell stage, so only half the embryo is perturbed. At tailbud stage 22, *in situ* hybridization was performed to the *twist* gene; *twist* is a marker for craniofacial neural crest cells (top panel control embryo/CE, 100% normal; *n* = 30). In middle and bottom panel, left side is injected and right side is normal. *Twist* expression is inhibited and (asymmetric, 60%; *n* = 40) in the left panel (*ASXL3-MO*), but its expression is normal on the uninjected side of the embryo in the right panel. **(C)** Embryos were injected with *ASXL3-MO* (12 ng) at the one-cell stage. Embryos were stained for cartilage formation at tadpole stages. Control embryos (90%, *n* = 27; panel) normal head cartilage staining (upper panel, left: head shot; right: dissected head cartilage). All morphant embryos (100%, *n* = 14) had reduced and abnormal head cartilage staining (lower panel, left: head shot; right: dissected head cartilage). **(D,E)** *In situ* hybridization was performed with a *Xenopus laevis ASXL3* probe to neurula (**D**; dorsal view, anterior-posterior is top-bottom) and tadpole stage 35 **(E)** embryos.

We also examined expression of anterior neural markers in ASXL3 morphant embryos. Interestingly, anterior neural expression in whole embryos recapitulated many of the observations seen in the previous AC experiment shown in Figure 4A. Expression of the most anterior forebrain marker, *xanf* is not significantly altered by ASXL3 knockdown (Figure 4C, lanes 2–4). However, expression of the forebrain specific *foxd1* and *foxg1* markers as well as the cement gland marker *xag* is highly reduced, whereas expression of the fore-midbrain marker *otx2* is only moderately perturbed (Figure 4C, lanes 2–4). The same was true for panneural markers, where expression of *nrp1* was fairly normal, but *ncam* expression was highly reduced (Figure 4C, lanes 2–4). As previously shown (Figure 1), expression of hindbrain (*krox20*, *hoxb3*), primary neuron (*ntub*) and neural crest (*snail2, foxd3*) markers was highly inhibited, but expression of the *cdx* genes in the spinal cord was normal or slightly increased (Figure 4C, lanes 2–4). Tailbud stage ASXL3 morphant embryos have a squat-body and perturbed head/trunk formation (Figure 5A), which is similar to phenotypes in which hindbrain and/or neural crest formation is perturbed (Dibner et al., 2001; Gutkovich et al., 2010).

The effects of ASXL3 knockdown are specific for neural tissue and are not a secondary result of a loss of mesoderm that regulates neural induction and patterning. Expression of dorsal (*gsc*, *xnot*, *chd*), dorsal-lateral (*myod*), ventral (*osr1/2*, *vent2*), and pan-mesodermal (*xbra*) markers in gastrula stage ASXL3 morphant embryos is normal (Figure 4E, compare lanes 2–4). In later stage sibling embryos, neural marker expression (*en2*, *krox20*, *ncam*, *ntub*, *foxd3*) is characteristically reduced (Figure 4F, compare lanes 2–4), while some markers are typically unaffected (*cdx2/4*, *sox2/3*). *Sox2/3* expression suggests that neural progenitor cell formation is not disrupted in the knockdown embryos (Figure 4F).

In ASXL3 morphants, we see abnormal head formation (Figure 5A), suggesting a disruption of more anterior neural tissue. The neural crest also seems atypical as seen by abnormal melanocyte patterning along the body axis and malformation of the dorsal fin (Figure 5A). In tailbud stage embryos, there is a strong reduction of *twist* marker expression (Figure 5B), which is specific for head/craniofacial neural crest cells (Figueiredo et al., 2017). Additionally, neural crest derived head cartilage formation is perturbed and reduced in ASXL3 morphant embryos (Figure 5C). The phenotypes seen in neurula (Figures 1, 2) through tadpole stage embryos (Figures 5A–C) are supported by the *ASXL3* gene expression patterns. By *in situ* hybridization (Figures 5D,E), *ASXL3* mRNA transcripts are detected throughout the neural plate in neurula stage embryos (Figure 5D) and predominantly in the head and dorsal fin in later tadpole stages (Figure 5E), regions derived from neural crest. *ASXL3* transcripts were not significantly detected in the more anterior cement gland or in the developing eye regions (Figure 5E).

ASXL3 knockdown causes a multitude of differential AP patterning disruptions in nervous system development. In the posterior nervous system, hindbrain, primary neuron and neural crest formation is perturbed, yet the spinal cord appears normal. Neural marker expression in the more anterior forebrain is also perturbed, but differentially and even panneural markers are not affected equally. These results suggest that ASXL3 acts downstream to a number of signaling pathways and transcription factors to differentially modulate chromatin in genes that respond to these cues. Perhaps, only a subset of neural-specific genes are affected in differing embryonic regions along the neural AP axis, which could be the trigger of the general nervous system defects seen in BRS.

## Discussion

In this study, we utilized *Xenopus laevis* as a platform to better understand the etiology of BRS during early embryonic development. BRS is caused by different truncations of the ASXL3 polycomb protein, and is heterozygous in nature. ASXL3 plays a role in H2A deubiquitination by binding the BAP1 protein to form the PR-DUB complex. This complex modifies H2A ubiquitination to regulate chromatin structure and gene expression. Indeed, in fibroblast cells derived from BRS patients, H2A ubiquitination was increased and over five-hundred genes had altered gene expression levels (Srivastava et al., 2016).

Our initial goal was to examine how *ASXL3-MO* protein knockdown modulates early nervous system development. In embryos, we found a differential inhibition of posterior neural cell fates. Hindbrain, neural crest and primary neuron cell fates were perturbed in neurula through tadpole stage embryos. The embryos also had impeded neural plate elongation and folding which seem to be partially compensated for at later stages. The spinal cord seemed to form normally and ASXL3 morphant embryos had slightly increased expression of some spinal cord markers. Our experiments in explants suggest that there is a differential requirement for ASXL3 protein in the hindbrain versus spinal cord. It is clear that while not identical to BRS, the perturbation of neural crest formation in *Xenopus* development may give us clues as to the initial etiology of the craniofacial defects found in BRS (Bainbridge et al., 2013; Dinwiddie et al., 2013; Russell and Graham, 2013; Hori et al., 2016; Balasubramanian et al., 2017; Kuechler et al., 2017; Bacrot et al., 2018). Formation of hindbrain, primary neurons and neural crest tissues are perturbed in ASXL3 morphant embryos. While BRS patients do not have an apparent strong knockdown/out phenotype, they do display similar phenotypic defects in craniofacial, motor neuron and hindbrain organization (Bainbridge et al., 2013; Dinwiddie et al., 2013; Russell and Graham, 2013; Hori et al., 2016; Balasubramanian et al., 2017; Kuechler et al., 2017; Bacrot et al., 2018). Our experiments in embryos in explants also show that the loss of ASXL3 activity modulates initial neural induction by modulating early expression of anterior and panneural markers induced by BMP antagonism. Anterior forebrain marker expression is variably disrupted in the ASXL3 morphant embryos, suggesting that in the forebrain ASXL3 acts to differentially regulate levels of transcription factors crucial for correct forebrain development. In ASXL3 morphants, the increase in *xanf1* expression could trigger repression of other transcription factor genes required for proper forebrain formation (Ermakova et al., 1999). It is interesting that RA signaling appears unaffected by ASXL3 knockdown. RA triggers activation of the retinoic acid receptor (RAR) that binds RARE elements in target genes during nervous system development (Janesick et al., 2015). Presumably, these elements are not irreversibly modified by ASXL3 mediated chromatin changes, and RAR proteins act independently of ASXL3 to bind RARE elements and activate RA-target gene expression. Further experimentation needs to be carried out to examine how ASXL3 regulates other signaling pathways required for hindbrain, neural crest and primary neuron formation. FGF and canonical Wnt signaling are crucial for the induction and specification of these cell types (Dorey and Amaya, 2010; Elkouby and Frank, 2010; Gutkovich et al., 2010). We will need to examine how ASXL3 protein knockdown modulates these signaling pathways and how this effects gene expression and the specification of these early posterior neural cell fates.

The genetic etiology of BRS is unknown, but interestingly both full-length and mutant/truncated ASXL3 proteins rescued the ASXL3 knockdown phenotype, by re-activating expression of hindbrain, neural crest and primary neuron marker genes. This observation suggests that at least in the assay utilized, the truncated protein is not dead or dominant-negative. It could be hypomorphic, and the forced ectopic levels of expression could circumvent this problem. Early *Xenopus* embryos are an excellent experimental system to investigate whether the ASXL3 mutation causes a dominant-negative, hypomorph or haploinsufficiency phenotype. We have already developed embryo and explant assays to address ASXL3 function in normal development. In future experiments, we can inject the *ASXL3-MO* at low to high concentration ranges in order to induce hypomorphic phenotypes in embryos/explants at the lower MO concentrations. The hypomorph concentrations can mimic the BRS disease phenotypes induced by haploinsufficiency as a result of RNA mediated decay or the mutant/truncated ASXL3 protein being a hypomorph/null protein. We can co-express full-length and mutant ASXL3 proteins in our various assays on the morphant background. *Xenopus ASXL3-MO* embryos/explants co-expressing equal levels of the wild-type and mutant proteins could mimic the mechanism of BRS. Haploinsufficiency is a possible cause of BRS (Srivastava et al., 2016). For ASXL2 truncation mutations, the mRNA does not undergo nonsense mediated decay, suggesting but not proving that that the protein is acting in a dominant-negative manner (Shashi et al., 2016). Depending on our accumulated knowledge, we will decide which of these approaches will be the best platform for transcriptome analysis versus control embryos. *Xenopus laevis* is unique in offering the resolution of a changing organism with a disease phenotype/state and not a cell line to examine this question. Via the *Xenopus* embryo, these future experiments identifying the earliest expressed embryonic target genes modulated by ASXL3 knockdown could be the key to understanding the embryonic etiology of BRS in humans.

Besides the ASXL3 truncation mutations associated with BRS, genomic studies have uncovered missense *ASXL3* variants linked to ASD that lack the BRS phenotype. How these mutant ASXL3 proteins cause BRS or missense variant proteins participate in ASD is not understood. Thus, another future goal is to develop bioassay screens to address functional differences in wildtype ASXL3 versus protein variants causing BRS or ASD. Often, there are no available bioassays to screen functional differences between proteins causing diseases or syndromes versus the normal protein. *Xenopus* may offer a solution to this problem. Our system offers an opportunity to address real-time physiological function *in vivo* during early development to actually address how a “model” protein causing genetic neural disorders or autism modulates early cell fate decisions, gene expression and neuron formation in the developing brain. We can leverage this system to understand the changing activities of mutant proteins that trigger human disorders. By understanding the role of ASXL3 in normal neural development in our different explant and embryo assays, we will substitute the various mutant versions (truncated or missense versions associated with BRS or ASD) of the protein and assay them for specific functions. Using these techniques, we will develop bioassays based on embryological activity to identify different activities of the ASXL3 protein mutants and missense-isoforms in involved in BRS or ASD. Thus, we hope to utilize the *Xenopus* embryonic system as a powerful tool to understand the molecular regulation of human genetic neural developmental disorders.

## Materials and Methods

### *Xenopus* Embryos and Explants

Ovulation, *in vitro* fertilization, culture, explant dissection and treatment were as described (Re’em-Kalma et al., 1995; Bonstein et al., 1998). Embryos were stained with Alcian Blue for cartilage detection at tadpole stages (Figueiredo et al., 2017).

### RNA and MO Injections

Both the full-length and the truncated-mutant ASXL3 pcDNA3.1/V5-His-TOPO vector constructs used for making mRNA were described in Srivastava et al. (2016). Capped sense *in vitro* transcribed mRNA constructs of human full-length and mutant-truncated *asxl3*, *meis3*, *noggin*, and *foxd1* (Salzberg et al., 1999; Srivastava et al., 2016; Polevoy et al., 2017) were injected into the animal hemisphere of one-cell stage embryos, as was the antisense translational-blocking *ASXL3*-morpholino oligonucleotide (*ASXL3-MO*; GeneTools) 5′GTCTTTCATGTTTGCATCTCATTGA -3′.

### *In situ* Hybridization

Whole-mount *in situ* hybridization was performed (Harland, 1991) with digoxigenin-labeled probes *neural-specific tubulin* (*n-tub*), *krox20*, *cdx4, twist* (Gutkovich et al., 2010; Fonar et al., 2011; Figueiredo et al., 2017), and *asxl3*. Two *Xenopus asxl3* probes were used. One was synthesized based on the *Xenopus asxl3* gene sequence (Syntezza Ltd.). The other probe was subcloned into a T-easy vector (Promega) from neurula stage mRNA by RT-PCR. Both probes gave identical *in situ* hybridization patterns. Control sense probes gave no specific expression pattern.

### Semi-Quantitative (sq) RT-PCR Analysis

sqRT-PCR was performed (Snir et al., 2006). In all sqRT-PCR experiments, three to six independent experimental repeats were typically performed. In all experiments, samples are routinely assayed a minimum of two times for each marker. sqRT-PCR Primers: efα, *odc*, *ncam*, *nrp1*, *sox2*, *sox3*, *xanf1*, *foxd1*, *foxg1*, *otx2*, *xag1*, *en2*, *krox20*, *hoxd1*, *hoxb1*, *hoxa2*, *hoxb3*, *hoxb4*, *hoxb5*, *RARα2.2*, *cdx1*, *cdx2*, *cdx4*, *n-tub*, *foxd3*, *slug* (*snail2*), *gsc*, *xnot*, *chd*, *myod*, *osr1*, *osr2*, *vent2*, and *xbra* (Aamar and Frank, 2004; Fonar et al., 2011; Elkouby et al., 2012; Bin-Nun et al., 2014).

## Ethics Statement

This study has been carried out under the guidelines of DF’s animal ethics permit (IL-134-11-18). The above research proposal has been reviewed by the Animal Care and Use Committee of the Technion, Israel Institute of Technology, and found to confirm with the regulations of this institution for work with laboratory animals. The Technion holds a valid Assurance (#A5027-01) of the US Department of Health & Human Services for humane care and use of laboratory animals.

## Author Contributions

HL, AA, HP, CR, SB, and DF conceived and designed the research. HL, AA, and HP performed the experiments. HL, AA, HP, and DF wrote the manuscript with input from CR and SB.

## Conflict of Interest

The authors declare that the research was conducted in the absence of any commercial or financial relationships that could be construed as a potential conflict of interest.
